# Characterization of a Methanogenic Community within an Algal Fed Anaerobic Digester

**DOI:** 10.5402/2012/753892

**Published:** 2012-06-21

**Authors:** Joshua T. Ellis, Cody Tramp, Ronald C. Sims, Charles D. Miller

**Affiliations:** Department of Biological Engineering, Utah State University, 4105 Old Main Hill, Logan, UT 84322, USA

## Abstract

The microbial diversity and metabolic potential of a methanogenic consortium residing in a 3785-liter anaerobic digester, fed with wastewater algae, was analyzed using 454 pyrosequencing technology. DNA was extracted from anaerobic sludge material and used in metagenomic analysis through PCR amplification of the methyl-coenzyme M reductase *α* subunit (*mcrA*) gene using primer sets ML, MCR, and ME. The majority of annotated *mcrA* sequences were assigned taxonomically to the genera *Methanosaeta* in the order *Methanosarcinales*. Methanogens from the genus *Methanosaeta* are obligate acetotrophs, suggesting this genus plays a dominant role in methane production from the analyzed fermentation sample. Numerous analyzed sequences within the algae fed anaerobic digester were unclassified and could not be assigned taxonomically. Relative amplicon frequencies were determined for each primer set to determine the utility of each in pyrosequencing. Primer sets ML and MCR performed better quantitatively (representing the large majority of analyzed sequences) than primer set ME. However, each of these primer sets was shown to provide a quantitatively unique community structure, and thus they are of equal importance in *mcrA* metagenomic analysis.

## 1. Introduction

Global energy requirements are heavily dependent on fossil fuels such as oil, coal, and natural gas. With the anticipation of fossil fuels being exhausted in the future, novel strategies need to be discovered for alternative energy generation. Of increasing importance is biogas production from renewable biomass feedstocks. The Logan City Wastewater Lagoon System (LCWLS) is an open-pond wastewater treatment facility that supports the growth of microbial communities that work symbiotically to metabolize and stabilize organic matter [1]. The microbial community present within the anaerobic sludge sediment has been used as inoculum for pilot scale anaerobic digestion processes where algal biomass is used as substrate. Algal biomass that occurs naturally in the LCWLS has been effectively harvested from the wastewater effluent and used for methane generation. Algae have been identified as a promising renewable energy feedstock due to their effective conversion of solar energy to biomass [2], which occurs naturally in this open-pond wastewater treatment facility. Anaerobically digested algal biomass generated from this system provides an appropriate technological approach to algal biofuels [3]. To date, methanogenic *Archaea* community-based studies on algal fed anaerobic digesters inoculated with wastewater sludge sediment have not been reported in the referred literature.

Anaerobic digestion is a series of processes in which microorganisms metabolize and stabilize biodegradable material in anaerobic conditions. These microbial interactions are considered to be symbiotic or even commensalistic interactions involving hydrolysis, acidogenesis, acetogenesis, and methanogenesis [1]. The process of anaerobic digestion is used for industrial or domestic purposes to manage waste and/or to release energy in the form of methane gas [4]. Methane is generated through anaerobic fermentation of low-molecular-weight carbon compounds through the process of methanogenesis [5]. Methanogenic *Archaea* play an essential role in the recycling of carbon in the biosphere and are estimated to produce approximately one billion tons of methane annually in anoxic conditions [6, 7], thus driving the motivation to employ this unique methanogenic physiotype at industrial scales. Methane derived from anaerobic treatment of organic wastes has a great potential to be an alternative fuel source and may stimulate independent and domestic energy economies [8, 9].

Diverse consortiums of methanogenic *Archaea* produce methane in the anaerobic sediments of the Logan Lagoons. These archaeal communities have not been studied to date, thus providing an uncultured archaeal community for study. There is increasing interest in analyzing the organization and function of biogas producing ecosystems, particularly since the relationships among biogas producing microbial populations are not well understood [5].

**Figure 1:**
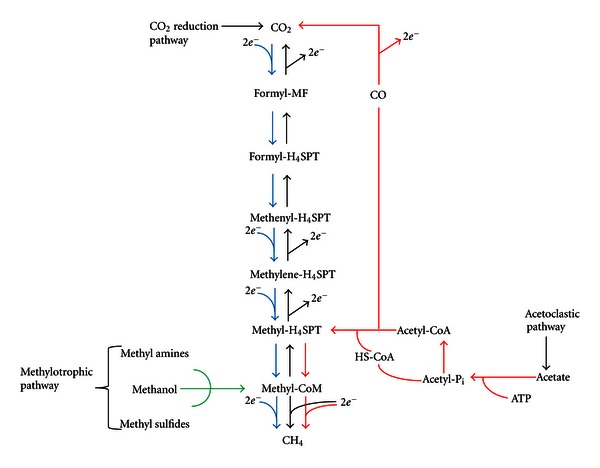
Schematic illustrating the major substrates (H_2_/CO_2_, methanol, methyl amines, methyl sulfides, and acetate) and the respective pathways utilized for methanogenesis (modified from [15, 30]).

Methanogenic *Archaea *is one of the largest and most phylogenetically diverse groups of microbes in the *Archaea* domain. Presently, six different orders of methanogens have been recognized: *Methanosarciniales, Methanomicrobiales, Methanococcales, Methanobacterales, Methanocellales, *and *Methanopyrales* [10, 11]. These microbes have evolved pathways for the metabolism of simple carbon substrates, such as acetate, carbon dioxide, formate, and methanol. There are generally three methanogenic pathways described throughout the literature. These pathways, shown in Figure 1, are as follows: (1) the CO_2_ reduction pathway involves the reduction of CO_2_ to CH_4_ with hydrogen gas as electron donor (hydrogenotrophic) and/or formate; (2) the methylotrophic pathway involves the disproportionation of methylated compounds, such as methanol and methylamines to CO_2_ and CH_4_; (3) the acetoclastic pathway involves the dismutation of acetate to CO_2_ and CH_4_ [12–15].

The methyl-coenzyme M reductase (MCR) is a holoenzyme that is composed of two alpha (*mcrA*), two beta (*mcrB*), and two gamma (*mcrG*) subunits, encoded by the *mcrBDCGA *operon. It catalyzes heterodisulfide formation and subsequent release of methane by combing the hydrogen donor coenzyme B and methyl donor coenzyme M [16, 17]. This enzyme is commonly referred to as isoenzyme MCRI. Additionally, members of the orders *Methanobacteriales* and *Methanococcales *carry the isoenzyme MCRII, coded by the *mrtBDGA *operon [18, 19]. MCR subunits are phylogenetically conserved throughout all methanogens and are necessary for the production of cellular energy. This protein is not found in bacteria, eukarya, or other *Archaea* [17]. Additionally, lateral gene transfer of MCR genes throughout *Archaea* species has not been observed [16], thus the MCR operon, and particularly the *mcrA* gene, has been widely used as an explicit marker for the detection of methanogenic diversity within a particular ecological niche [5, 11, 19–22].

To date, there is no refereed literature regarding methanogenic communities inhabiting algal fed anaerobic digesters. However, there are several descriptions of biogas production using algal biomass as substrate [2, 23–27]; yet, no information on methanogenic consortiums in these systems exists to date. To advance the understanding of methanogenic consortiums inhabiting an algal fed anaerobic digester inoculated with anaerobic sludge material from the LCWLS, metagenomic analysis of the methyl-coenzyme M reductase alpha subunit (*mcrA*) gene was carried out using 454 pyrosequencing. Pyrosequencing technology has provided the ability to efficiently sequence target genes from environmental samples, while overriding cloning biases and sequence limitation from traditional clone libraries [5].

Three primer sets, ML, MCR, and ME (Table 1), have been described previously for comparing methanogenic *Archaea *communities in *mcrA* clone libraries [11, 19], but have not been reported to be incorporated into high-throughput 454 pyrosequencing in combination to determine the feasibility of these primers in methanogenic *Archaea *metagenomic analysis. In order to accurately demonstrate the diversity of an environmental sample, pyrosequencing technologies can be employed. Pyrosequencing allows investigators to examine thousands of sequences, while allowing the discovery of rare organisms among thousands of dominant species, both of which are extremely difficult in clone library methodologies. Recently, pyrosequencing of a biogas microbial community within a maize silage, green rye, and liquid manure fed anaerobic digester using primer sets ML and ME described the taxonomic order *Methanomicrobiales* and, more particularly, *Methanoculleus bourgensis, *as being the dominant species within the analyzed fermentation sample [5].

This study focuses on the structure and characterized diversity of a methanogenic consortium and its metabolic potential residing in biosolids sediment within a 3785-liter algal fed anaerobic digester, with emphasis on the *mcrA *gene using and analyzing primer sets ML, MCR, and ME.

## 2. Methods

### 2.1. Sampling Site and Characteristics 

Sludge material from a 3785-Liter algal fed anaerobic digester was collected from a sampling port on the bottom of the digester and immediately stored under N_2_. This anaerobic digester was operated at 37°C, had a hydraulic retention time of 20 days, and operated in fed batch mode. Algae substrate was harvested from lagoon wastewater effluent using a dissolved air floatation unit, with an average concentration of 10 g L^−1^.

### 2.2. Nucleic Acid Extraction and Amplification of *mcrA* Genes

**Figure 2:**
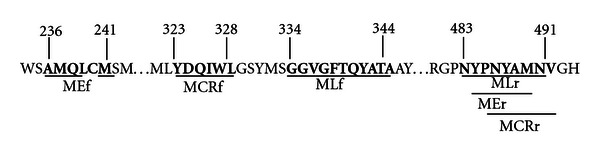
Target sites for *mcrA *primers according to McrA sequence of *Methanosaeta concilii *GP-6 (YP_004383383.1). Primer MEf does not have complementary base pairing to this particular sequence, as shown by a gap under amino acids leucine (L) and cysteine (C). Amino acid sequences are presented to illustrate the degeneracy based on amino acid codon differences.

Total community DNA was extracted from 250 mg of sludge sediment using the PowerSoil DNA Isolation Kit (MO BIO Labs. Inc., Solana Beach, CA). Samples were stored under N_2_ for no longer than 30 minutes prior to DNA isolation. The degenerate archaeal primers, ML, MCR, and ME [11, 21, 28], were used to PCR-amplify *mcrA *gene fragments from purified DNA (Table 1). These primer sets have partially overlapping target regions as shown in Figure 2. Primers sequences were as follows (5′-3′): MLf: GGTGGTGTMGGATTCACACARTAYGCWACAGC, MLr: TTCATTGCRTAGTTWGGRTAGTT [19, 29], and previously described as primer Luton *mcrA* [11, 21]; MCRf: TAYGAYCARA THTGGYT, MCRr: ACRTTCATNGCRTARTT  [11, 19]; MEf: GCMATGCARAT HGGWATG TC; MEr: TCATKGCRTAGTTDGGRTAGT [11, 19, 20, 28]. Appropriate tags and multiplex identifiers were used for each primer set for downstream 454 pyrosequencing. The PCR mixture contained 1 *μ*L of DNA (25 ng final concentration for reactions concerning primer ML and MCR, and 40 ng concerning primer ME), 1 *μ*L of each primer (25 *μ*M), 5 *μ*L of 10x PCR buffer, 1 *μ*L of bovine serum albumin (15 mg/mL), 5 *μ*L of deoxynucleoside triphosphates (2 mM each of dATP, dTTP, dGTP, and dCTP), 0.5 *μ*L *Taq* DNA polymerase (5U/*μ*L), and 2.5 *μ*L MgCl_2 _(25 mM) in a final reaction volume of 50 *μ*L. Amplification was carried out as follows: initial denaturation for 2 min at 95°C, 35 cycles of 95°C for 1 min, annealing at 58°C (ML), 50°C (MCR), or 56°C (ME) for 1 min, and 1.5 min at 72°C, with a final extension for 12 min at 72°C. PCR products were checked for positive amplification and correct amplicon size by agarose gel electrophoresis. Positive amplicons were purified using the PCR purification kit (Qiagen Inc., Valencia, CA), as indicated by the manufacturer. Target PCR amplicons were of sizes 470 bp, 500 bp, and 760 bp for primer sets ML, MCR, and ME, respectively, (Table 1).

### 2.3. High-Throughput Sequencing and BLASTn Analysis of *mcrA* Metagenome Reads 

Sequencing runs were performed on *mcrA* libraries prepared from total sludge community DNA using the Roche Genome Sequencer (GS) FLX System. Samples were pooled together and incorporated into two wells of the 454 plate. The sequencing data output file was analyzed using a program written in Visual Basic.NET. This program converted the  .fna data file into standard FASTA format. Sequences were sorted by primer ID tags, ID tags were removed, and sequences were filtered by length. Only sequences over 100 bp in size were analyzed by BLASTn; sequences shorter than 100 bp were mainly primer dimer reads and were thus redundant in this analysis. Identical sequences were combined, and all sequences were named with the primer set code, an ID number, and the number of combined sequences it represented. BLASTn analysis was conducted using the “Nucleotide collection (nr/nt)” database and allowing 20,000 max target sequences. For each sequence, a list of top BLAST hits was compiled, filtered for alignments of at least 50 bp and *E*-values smaller than 1*e* ^−6^. Uncultured clones were removed from the list of top hits to allow us to derive information on the functionality of the system in the sense of metabolic potential and community structures based on characterized physiotypes. For each sequence, the BLAST hit with the highest BLAST score was selected as the match's species. Sequence similarities were all greater than 97% identical to the species identified. A total of each species' hit count was then generated, taking into account the number of identical sequences that were combined into each analyzed sequence prior to BLASTn analysis.

### 2.4. Phylogenetic Analysis

Nucleotide sequences for *mcrA* genes were pooled together for each primer set and used to determine the phylogenetic diversity. Phylogenetic analysis of *mcrA* sequences was accomplished by using the MEGA 5.01, Molecular Evolutionary Genetic Analysis web-based software package [31, 33]. Alignment files were generated using ClustalW, a function within MEGA. The evolutionary history was inferred by using the Maximum Likelihood method based on the Tamura-Nei model [33]. Phylogenetic trees with the highest log likelihood are shown. The percentage of trees in which the associated taxa clustered together is shown next to the branches. Initial tree(s) for the heuristic search were obtained automatically as follows. When the number of common sites was less than 100, or less than one-fourth of the total number of sites, the maximum parsimony method was used; otherwise BIONJ method with MCL distance matrix was used. The tree is drawn to scale, with branch lengths measured in the number of substitutions per site [31, 33].

## 3. Results and Discussion

### 3.1. Methanogenic Community Structure Analysis Based on High-Throughput 454 Sequencing of Methyl-Coenzyme M Reductase Genes

**Figure 3:**
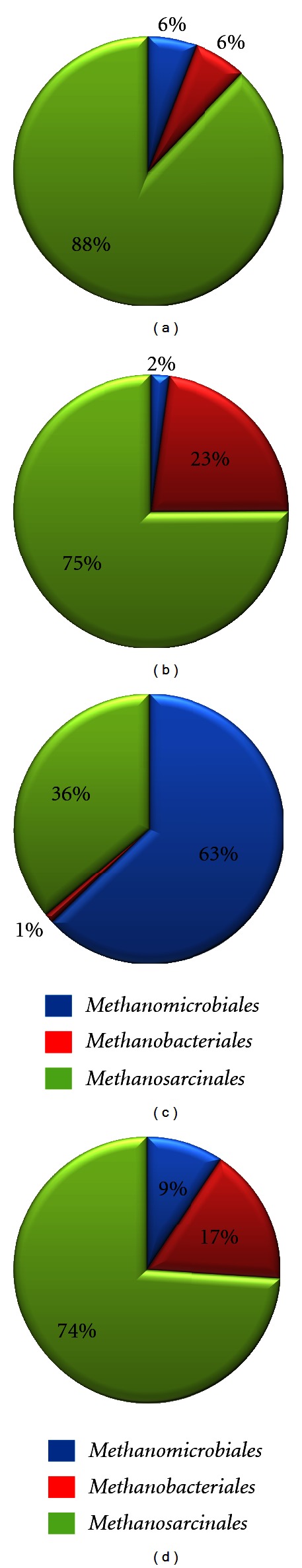
Taxonomic classification of *mcrA* 454 sequences. (a) Primer ML. (b) Primer MCR. (c) Primer ME. (d) All primers. Only assignments with *E*-values smaller than 1*e* ^−6^ were used in this assessment. *mcrA* sequences were assigned to the taxa level order.

Community DNA extracted from a fermentation sample was evaluated using three *mcrA-*specific primer sets (ML, MCR, and ME). This study provided useful information on the effectiveness of these primers in metagenomic analysis. Additionally, phylogenetic analysis as well as the metabolic potential of the anaerobic system was established from 454 pyrosequencing data. Purified DNA was used as template for PCR-based amplification of community *mcrA *genes. Positive amplicons were employed in high-throughput 454 pyrosequencing and analyzed as described above. Pyrosequencing output files described a total of 57,758 total sequences. 454 sequences less than 100 bps (shown to be primer dimer formation) were removed from the analysis to prevent redundancy. Analysis of *mcrA *gene sequences using the BLAST-nr database (*E*-value < 1*e* ^−6^) designated 1,634 sequences to match known or characterized methanogens, all of which had sequence similarity of at least 97% and *E*-value < 1*e* ^−6^. This was performed to allow relevant information to be derived on the functionality of biogas production from the algae fed anaerobic digester. After filtering data sets as described above, primer ML had a total of 382 *mcrA *sequences, primer MCR had a total of 1,080 sequences, and primer ME had a total of 172 *mcrA *sequences. A total of 1,634 methyl-coenzyme M reductase genes was incorporated into the final analysis. Sequence data from each primer set and pooled data were then organized taxonomically on order (Figure 3), genus, and species (Table 2). About 14% of analyzed sequences could not be assigned taxonomically, described as no significant similarity found (Table 2), indicating that many of the methanogens within the algal fed anaerobic digester are unclassified or novel. Only 1,634 sequences were analyzed in this study due to the removal of many thousands of uncultured or uncharacterized clone sequences which currently do not provide any useful information on the functionality of the system, however, indicate that isolation and characterization of these methanogens would provide a more comprehensive understanding of the system.

### 3.2. *mcrA* Primer Analysis

The molecular approach described above has identified various unique sequences among primer sets ML, MCR, and ME. Despite multiple attempts to optimize PCR conditions, the low efficiency of primer set ME gave poor yields of PCR products compared to primer sets ML and MCR, as determined by analysis of agarose gel band intensity and spectrophotometric measurements of purified PCR products. The ME primer set may not have been ideal for proper annealing with the large majority of methanogenic *mcrA *genes in our community DNA samples (as shown in Figure 2). The ME primer set has been described to capture a wide range of methanogens, but our community composition was strongly dominated by members of the order *Methanosarcinales*, in which primer set ME has shown difficulties in amplifying [34]. However, all primer sets with 454 tags were able to positively amplify *mcrA *genes within the representative sample for downstream pyrosequencing.

Analysis of the community composition depicts molecular bias towards amplification of *mcrA *gene fragments, which frequently occurs with PCR-based methods. The utilization of degenerate primers (Table 1) targeting a functional gene is subject to molecular bias due to the degeneracy of the genetic code [11]. The vast majority of the species discovered using primer sets ML and MCR were *Methanosaeta concilii;* however no hits on these genera were observed using primer set ME. Figure 2 provides additional evidence as MEf did not have ample complementary base pairing, as shown when analyzing Table 1 and Figure 2. The *Methanogenic* community sets based taxonomically on order represent the molecular bias described, particularly between primer sets ML and MCR compared to primer set ME (Figure 3). Additionally, greater methanogenic diversity in our metagenomic library using primers ML and MCR was observed. The metagenomic library constructed from the ME primers had a reduced methanogenic diversity compared to the other primer sets used in our analysis. These data concur with preliminary work done in our research group where a *mcrA *clone library was constructed using the same primer sets (data not shown). Additionally, this trend is somewhat consistent throughout the literature where the ME primer set provides noticeably less diversity in the context of *mcrA *libraries [11, 19], but is still valuable in identifying unique community structures.

### 3.3. Phylogenetic Analysis of the Algal Fed Sludge Metagenome

 Metagenomics has provided more accurate estimations of microbial diversity within environmental samples compared to clone libraries, where multiple biases exist along with sequencing limitations. Additionally, metagenomics is aimed at obtaining an unbiased view of community consortiums within a particular environment [5]. Although an abundance of short amplicon sequences occur in pyrosequencing, the ability to discover the presence of a species that is substantially less abundant than others within a microbial consortium is demonstrated. The sensitivity of pyrosequencing has allowed several organisms that were only present once out of 1,634 characterized sequences to be identified, whereas the probability of locating these rare species amongst a consortium of microbes using clone libraries would be very low.

**Figure 4:**
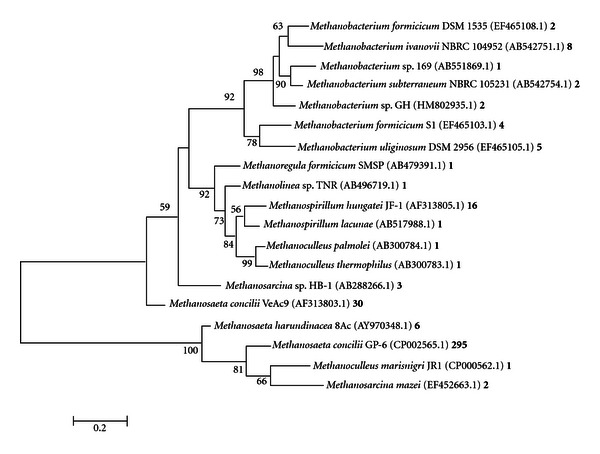
Phylogenetic analysis of *mcrA* sequences developed from primer ML. Phylogenetic tree was constructed using MEGA 5.01 Molecular Evolutionary Genetic Analysis web-based software package [31, 32]. This phylogenetic tree was generated using maximum likelihood analysis with 1000 bootstraps. Numbers at the nodes represent bootstrap values, with only values above 50 shown. Scale bar corresponds to 0.2 substitutions per nucleotide position. Accession numbers are shown in parenthesis. The number following the accession number represents the number of hits for that organism.

**Figure 5:**
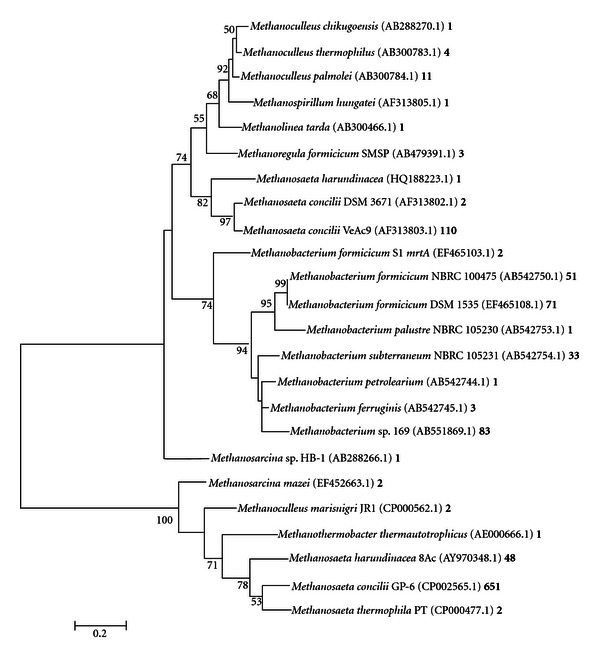
Phylogenetic analysis of *mcrA* sequences acquired from primer MCR. Phylogenetic tree was constructed using MEGA 5.01 Molecular Evolutionary Genetic Analysis web-based software package [31, 32]. This phylogenetic tree was generated using maximum likelihood analysis with 1000 bootstraps. Numbers at the nodes represent bootstrap values, with only values above 50 shown. Scale bar corresponds to 0.2 substitutions per nucleotide position. Accession numbers are shown in parenthesis. The number following the accession number represents the number of hits for that organism.

**Figure 6:**
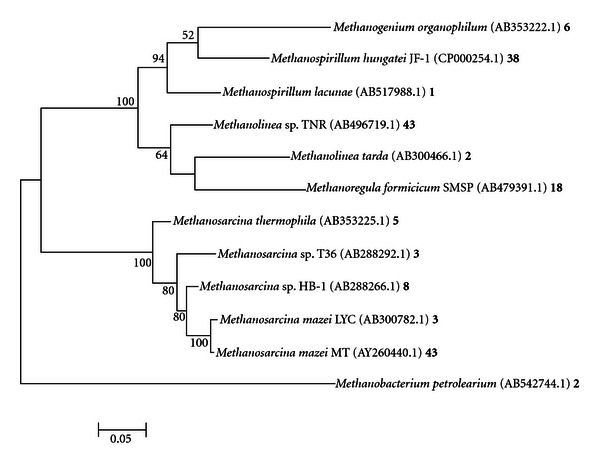
Phylogenetic analysis of *mcrA* sequences from primer ME. Phylogenetic tree was constructed using MEGA 5.01 Molecular Evolutionary Genetic Analysis web-based software package [31, 32]. This phylogenetic tree was generated using maximum likelihood analysis with 1000 bootstraps. Numbers at the nodes represent bootstrap values, with only values above 50 shown. Scale bar corresponds to 0.1 substitutions per nucleotide position. Accession numbers are shown in parenthesis. The number following the accession number represents the number of hits for that organism.

**Figure 7:**
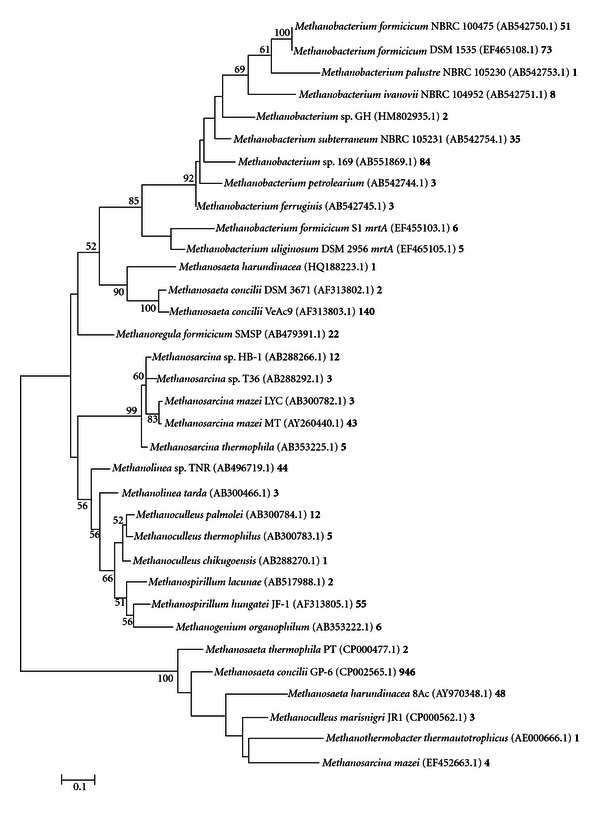
Phylogenetic analysis of *mcrA* sequences from pooled data sets from primers ML, MCR, and ME. Phylogenetic tree was constructed using MEGA 5.01 Molecular Evolutionary Genetic Analysis web-based software package [31, 32]. This phylogenetic tree was generated using maximum likelihood analysis with 1000 bootstraps. Numbers at the nodes represent bootstrap values, with only values above 50 shown. Scale bar corresponds to 0.1 substitutions per nucleotide position. Accession numbers are shown in parenthesis. The number following the accession number represents the number of hits for that organism.

The phylogeny of methanogenic *Archaea *from primer sets ML, MCR, and ME is depicted in Figures 4, 5, and 6, respectively. The vast majority of organisms displayed in the phylogenetic tree from Figure 7 were from ML and MCR data sets. This is consistent with the analysis from Figure 3 in that the relative amplicon frequencies from primer ML, MCR, and the pooled data represent methanogens from the order *Methanosarcinales*. Primer set ME did not generate any hits from the genera *Methanosaeta*, which represented 71% of the total sequences. However, this primer depicted novel microbes that were not represented with the other primers. Juottonen et al., 2006 [19] described faults with primer ME in amplifying members of the order *Methanosarcinales*, concurring with our overall analysis of primer ME and its output data. These phylogenetic trees which comprise only characterized methanogens are to validate or derive relevant information on the functionality of the anaerobic system. Again, it is important to consider that there were many uncharacterized or uncultured clone methanogen sequences within this system; however uncharacterized clone sequences do not provide significant data on existing functionality.

Analysis of *mcrA *sequences from the algal fed anaerobic digester revealed a broad spectrum of methanogenic microbes. This phylogenetic analysis based on pyrosequencing provided adequate insight into the phylogenetic structure of our system since phylogenetics depicts evolutionary relationships and distances between given genetic fragments [5]. Descriptions of biogas production communities have been established using high-throughput 454 sequencing technologies [5, 35]. Kröber et al., 2009 [5] describe the taxonomic order *Methanomicrobiales *and, more particularly, *Methanoculleus bourgensis, *as being the dominant speices within a maize silage, green rye, and liquid manure fed anaerobic digester using primer sets ML and ME. Using primer sets ML, MCR, and ME, we have shown our algal fed anaerobic digester to be highly dominated by the order *Methanosarcinales *and the obligate acetoclastic genera *Methanosaeta*. Our phylogenetic relationship in this study would has been significantly skewed if primer set MCR was missing from the study.

### 3.4. Metabolic Potential of the Algal Fed Sludge Metagenome

 Approximately two-thirds of the methane produced in the biosphere is derived from the acetoclastic pathway [34]. Only two genera of methanogenic *Archaea*, *Methanosaeta *and *Methanosarcina*, have been isolated and identified as utilizing acetate for methanogenesis [34]. Approximately 74% percent of our pooled 454 data was dominated by the order *Methanosarcinales *(Figure 3), including the genera *Methanosarcina *and the highly dominant genera *Methanosaeta*. *Methanosaeta* are considered obligate acetotrophs, in that they solely use acetate for methanogenesis [34]. The genera *Methanosaeta *greatly dominated the methanogenic diversity suggesting that our particular mesophilic system may have a low concentration of acetate, favoring *Methanosaeta *spp., which have been recognized as having a subordinate threshold for acetate compared to other acetotrophs associated with the family *Methanosarcinaceae* [36].* Methanosarcina mazei *are capable of producing methane through all three pathways described (Table 2). However, only some strains of this genus can utilize H_2_/CO_2_ as substrates from methanogenesis [13]. *Methanosarcina thermophila *can utilize the acetoclastic and the methylotrophic pathways for methanogenesis [13, 36] (Table 2).

Members of the order *Methanobacteriales*, and particularly those belonging to the genera *Methanobacterium*, all use the CO_2_ reduction pathway with H_2_ as electron donor for methanogenesis [37]. Some species of this genus such as *M. formicicum* and *M. palustre* can also reduce CO_2_ to methane using formate as the electron donor. *Methanothermobacter thermautotrophicus* can also drive methanogenesis by utilizing the CO_2_ reduction pathway with H_2_ and/or formate as electron donor [13]. Several *mrtA *genes were detected from organisms *M. formicicum *S1 and *M. uliginosum *DSM 2956 using primers ML and MCR (Table 2). This gene is said to be predominately expressed when the H_2_ supply is not growth rate limiting, whereas *mcrA *would be formed when availability of H_2_ is limited. When H_2_ supplies are not limited, the MCR reaction may be the rate-limiting step in the methanogenesis pathway, thus it would be physiologically relevant to synthesize an enzyme with a higher **V*_max⁡_* [18].

From the order Methanomicrobiales, organisms *Methanoculleus palmolei*, *Methanoculleus marisnigri*, *Methanoculleus thermophiles*, *Methanoculleus chikugoensis* [13, 38], *Methanogenium organophilum* [13], *Methanolinea tarda* [39], *Methanoregula formicicum* [40], *Methanospirillum lacunae*, and *Methanospirillum hungatei* [13, 41], all of which are present in our anaerobic digester, are all capable of utilizing the CO_2_ reduction pathway with either H_2_/CO_2_, or formate as substrates. *M. palmolei* and *M. chikugoensis* can also utilize the methylotrophic pathway for methanogenesis by metabolizing secondary alcohols [38] (Table 2).

Of the total 1,634 methyl-coenzyme M reductase sequences analyzed, approximately 74% of the assigned methanogens could utilize the acetoclastic pathway, due to the high abundance of *Methanosaeta*. About 30% of the assigned methanogens were hydrogenotrophic, and 17% of the total methanogens could also reduce CO_2_ to CH_4_ with formate as the electron donor. In addition, about 56% of those methanogens that were hydrogenotrophic could reduce CO_2_ to CH_4_ with formate as the electron donor. Only about 4% of the total methanogens were methylotrophic. These results suggest that species relating to the genus *Methanosaeta* obligate acetotrophs, and members of the order *Methanosarcinales* play a dominant role in the production of CH_4_ in the algal fed anaerobic digester.

## 4. Conclusions

A comprehensive investigation of the phylogeny and metabolic potential of methanogenic *Archaea* residing in an algal fed anaerobic digester was accomplished using three different *mcrA *primer sets. The *mcrA *gene encodes the *α*-subunit of methyl-coenzyme M reductase and is widely used as a phylogenetic marker for characterization of methanogenic communities because it is conserved throughout all methanogenic *Archaea* [5, 21]. Although primer set ME exhibited deficiencies in amplifying *mcrA *genes from *Methanosarcinales *and depicted less methanogenic diversity compared to primer sets ML and MCR, it was a valuable tool in this analysis as it identified marginal methanogens that would have been absent otherwise. Juottonen et al., 2006 [19] described that the use of these three primer sets provided a quantitatively unique community structure through clone libraries, and they were confirmed to do so as well using pyrosequencing technology. Several hits only appeared once out of all the analyzed sequences, showing the sensitivity of high-throughput 454 sequencing technologies over standard clone libraries. Accordingly, the use of these three primer sets provided a comprehensive analysis of the methanogenic *Archaea* residing in an algal fed anaerobic digester, and these sets were found to all be of equal significance in *mcrA *metagenomic analysis. A large portion of the analyzed sequences could not be assigned taxonomically, signifying that many of the methanogens within the analyzed fermentation sample are unclassified or novel. Phylogenetic analysis of this algal fed anaerobic digester indicates a broad range of methanogens from the orders *Methanobacteriales, Methanomicrobiales, *and *Methanosarcinales*, with the latter being the overall dominant order. Additionally, these results suggest that species relating to the genus *Methanosaeta*, members of the order *Methanosarcinales, *which are obligate acetotrophs, play a dominant role in methanogenesis in the analyzed fermentation sample.

## Figures and Tables

**Table 1:** Primer sets used to amplify *mcrA *gene fragments.

Primer	Sequence 5^′^-3^′^	Amplicon size (bps)
ML	F: GGTGGTGTMGGATTCACACARTAYGCWACAGC	~470
	R: TTCATTGCRTAGTTWGGRTAGTT	
MCR	F: TAYGAYCARATHTGGYT	~500
	R: ACRTTCATNGCRTARTT	
ME	F: GCMATGCARATHGGWATGTC	~760
	R: TCATKGCRTAGTTDGGRTAGT	

**Table 2:** Comparison of *mcrA* metagenomic library sequences from sludge community DNA to analogous NCBI nucleotide sequence database records through BLASTn utilizing the nonredundant database and excluding uncultured/environmental sample sequences. Only hits with an *E*-value < 1*e* ^−6^ were used in the final analysis. Metabolism: (1) acetoclastic, (2) CO_2 _ reduction with H_2_ (hydrogenotrophic) and formate, and (3) methylotrophic pathways [13].

Hits	Organism	Order	Metabolism
946	*Methanosaeta concilii* GP-6 (CP002565.1)	*Methanosarcinales*	1
262	No significant similarity found	NA	NA
140	*Methanosaeta concilii* VeAc9 (AF313803.1)	*Methanosarcinales*	1
84	*Methanobacterium kanagiense* 169 (AB551869.1)	*Methanobacteriales*	2
73	*Methanobacterium formicicum* DSM 1535 (EF465108.1)	*Methanobacteriales*	2^∗^
55	*Methanospirillum hungatei* JF-1 (CP000254.1)	*Methanomicrobiales*	2^∗^
51	*Methanobacterium formicicum* NBRC 100475 (AB542750.1)	*Methanobacteriales*	2^∗^
48	*Methanosaeta harundinacea* 8Ac (AY970348.1)	*Methanosarcinales*	1
44	*Methanolinea* sp. TNR (AB496719.1)	*Methanomicrobiales*	2^∗^
43	*Methanosarcina mazei* strain MT (AY260440.1)	*Methanosarcinales*	1, 2, 3
35	*Methanobacterium subterraneum* NBRC 105231 (AB542754.1)	*Methanobacteriales*	2
22	*Methanoregula formicicum* SMSP (AB479391.1)	*Methanomicrobiales*	2^∗^
12	*Methanoculleus palmolei* (AB300784.1)	*Methanomicrobiales*	2^∗^, 3
12	*Methanosarcina* sp. HB-1 (AB288266.1)	*Methanosarcinales*	1, 2
8	*Methanobacterium ivanovii* NBRC 104952 (AB542751.1)	*Methanobacteriales*	2
6	*Methanobacterium formicicum* S1 *mrtA* (EF465103.1)	*Methanobacteriales*	2^∗^
6	*Methanogenium organophilum* (AB353222.1)	*Methanomicrobiales*	2^∗^
5	*Methanoculleus thermophilus* (AB300783.1)	*Methanomicrobiales*	2^∗^
5	*Methanosarcina thermophila* (AB353225.1)	*Methanosarcinales*	1, 2
5	*Methanobacterium uliginosum* DSM 2956 *mrtA *(EF465105.1)	*Methanobacteriales*	2
4	*Methanosarcina mazei* (EF452663.1)	*Methanosarcinales*	1, 2, 3
3	*Methanobacterium ferruginis* (AB542745.1)	*Methanobacteriales*	2
3	*Methanobacterium petrolearium* (AB542744.1)	*Methanobacteriales*	2
3	*Methanoculleus marisnigri* JR1 (CP000562.1)	*Methanomicrobiales*	2^∗^
3	*Methanolinea tarda* (AB300466.1)	*Methanomicrobiales*	2^∗^
3	*Methanosarcina mazei* LYC (AB300782.1)	*Methanosarcinales*	1, 2, 3
3	*Methanosarcina* sp. T36 (AB288292.1)	*Methanosarcinales*	1, 2, 3
2	*Methanobacterium* sp. GH (HM802935.1)	*Methanobacteriales*	2
2	*Methanosaeta concilii* DSM 3671 (AF313802.1)	*Methanosarcinales*	1
2	*Methanosaeta thermophila* PT (CP000477.1)	*Methanosarcinales*	1
2	*Methanospirillum lacunae* (AB517988.1)	*Methanomicrobiales*	2^∗^
1	*Methanobacterium palustre* NBRC 105230 (AB542753.1)	*Methanobacteriales*	2^∗^
1	*Methanoculleus chikugoensis* (AB288270.1)	*Methanomicrobiales*	2^∗^, 3
1	*Methanosaeta harundinacea* (HQ188223.1)	*Methanosarcinales*	1
1	*Methanothermobacter thermautotrophicus* (AE000666.1)	*Methanobacteriales*	2^∗^

2^∗^: organisms capable of utilizing both H_2 _ and formate as the electron donors for methanogenesis from CO_2_. Methanogens that only use H_2_/CO_2 _ (hydrogenotrophic) are denoted with a 2.
